# Supplementary material for: Mendelian randomization suggests a bidirectional, causal relationship between physical inactivity and adiposity
Source: eLife. 2022 Mar 7;11:e70386. doi: 10.7554/eLife.70386 (PMC8975550; doi:10.7554/eLife.70386)
Supplement: Transparent reporting form [file elife-70386-transrepform1.docx]

***eLife’s* transparent reporting form**

We encourage authors to provide detailed information *within their submission* to facilitate the interpretation and replication of experiments. Authors can upload supporting documentation to indicate the use of appropriate reporting guidelines for health-related research (see [EQUATOR Network](http://www.equator-network.org/%20)), life science research (see the [BioSharing Information Resource](https://biosharing.org/)), or the [ARRIVE guidelines](http://www.plosbiology.org/article/info:doi/10.1371/journal.pbio.1000412) for reporting work involving animal research. Where applicable, authors should refer to any relevant reporting standards documents in this form.

If you have any questions, please consult our Journal Policies and/or contact us: [editorial@elifesciences.org](mailto:editorial@elifesciences.org).

**Sample-size estimation**

- You should state whether an appropriate sample size was computed when the study was being designed
- You should state the statistical method of sample size computation and any required assumptions
- If no explicit power analysis was used, you should describe how you decided what sample (replicate) size (number) to use

Please outline where this information can be found within the submission (e.g., sections or figure legends), or explain why this information doesn’t apply to your submission:

In the Method section we indicate that we “We used summary results from the largest published genome-wide association studies”.We implemented various methods including CAUSE that improves power by including all genetic variants, we stated in the Methods “The recently published Causal Analysis Using Summary Effect Estimates (CAUSE) Mendelian randomization method (Morrison et al., 2020) improves statistical power in such cases, by utilizing full genome-wide summary results instead of genome-wide significant loci only”. Further in the Discussion section we stated that “Here, using a method that takes advantage of full genome-wide summary results and corrects for sample overlap between the exposure and the outcome traits to maximize statistical power and correct for pleiotropy”. We also acknowledge a limitation in the discussion that “even if we used the largest available data on objectively measured physical activity, the statistical power was limited, as very few genome-wide significant loci have thus far been identified.” The assumptioms of the Mendelian randomization methods are described in Figure 1.

**Replicates**

- You should report how often each experiment was performed
- You should include a definition of biological versus technical replication
- The data obtained should be provided and sufficient information should be provided to indicate the number of independent biological and/or technical replicates
- If you encountered any outliers, you should describe how these were handled
- Criteria for exclusion/inclusion of data should be clearly stated
- High-throughput sequence data should be uploaded before submission, with a private link for reviewers provided (these are available from both GEO and ArrayExpress)

Please outline where this information can be found within the submission (e.g., sections or figure legends), or explain why this information doesn’t apply to your submission:

How every analysis was performed and corresponding curated data is indicated in the “Data sharing” section where a code for replication can be found for each of the analyses performed in this study. Outlier removal is explain in the Methods and Appendix, for instance “We performed outlier extraction using RadialMR to control for uncorrelated pleiotropy.” Finally, in the Discussion we stated that “When larger sample sizes for accelerometer-based physical activity become available, the results should be replicated.”

**Statistical reporting**

- Statistical analysis methods should be described and justified
- Raw data should be presented in figures whenever informative to do so (typically when N per group is less than 10)
- For each experiment, you should identify the statistical tests used, exact values of N, definitions of center, methods of multiple test correction, and dispersion and precision measures (e.g., mean, median, SD, SEM, confidence intervals; and, for the major substantive results, a measure of effect size (e.g., Pearson's r, Cohen's d)
- Report exact p-values wherever possible alongside the summary statistics and 95% confidence intervals. These should be reported for all key questions and not only when the p-value is less than 0.05.

Please outline where this information can be found within the submission (e.g., sections or figure legends), or explain why this information doesn’t apply to your submission:

All Mendelian randomization methods and statistical tests are described under the Method section subtitles “Mendelian randomization using full genome-wide summary results for the exposure trait” and “Mendelian randomization using genome-wide significant loci for the exposure trait”. Details are explained in the Appendix 1 text sections “Mendelian randomization using the CAUSE method” and “Mendelian randomization using the IVW, Egger, weighted median, and weighted mode methods” Dispersion, precision measures and exact p-values can be found in Table 1-2 and also Figure 2 in the main text, and in the Supplementary Files 1-7.

(For large datasets, or papers with a very large number of statistical tests, you may upload a single table file with tests, Ns, etc., with reference to sections in the manuscript.)

**Group allocation**

- Indicate how samples were allocated into experimental groups (in the case of clinical studies, please specify allocation to treatment method); if randomization was used, please also state if restricted randomization was applied
- Indicate if masking was used during group allocation, data collection and/or data analysis

Please outline where this information can be found within the submission (e.g., sections or figure legends), or explain why this information doesn’t apply to your submission:

This is a Mendelian randomization analysis, thus not applicable.

**Additional data files (“source data”)**

- We encourage you to upload relevant additional data files, such as numerical data that are represented as a graph in a figure, or as a summary table
- Where provided, these should be in the most useful format, and they can be uploaded as “Source data” files linked to a main figure or table
- Include model definition files including the full list of parameters used
- Include code used for data analysis (e.g., R, MatLab)
- Avoid stating that data files are “available upon request”

Please indicate the figures or tables for which source data files have been provided:

All numerical data is shared in the main text, Appendix and supplements. Summary level data and code for replication is under the “Data sharing” section: “All analyses were performed using R statistical package freely available at https://cran.r-project.org/mirrors.html. The CAUSE R package and instructions are available at https://jean997.github.io/cause/. The Two-sample MR package is available at https://mrcieu.github.io/TwoSampleMR/. The RadialMR package is available at https://github.com/WSpiller/RadialMR. The code and curated data for the current analysis are available at https://github.com/MarioGuCBMR/MR_Physical_Activity_BMI.”
